# Identifying potential biomarkers of nonalcoholic fatty liver disease via genome-wide analysis of copy number variation

**DOI:** 10.1186/s12876-021-01750-4

**Published:** 2021-04-14

**Authors:** Yang fan Li, Jing Zheng, He wei Peng, Xiao lin Cai, Xin ting Pan, Hui quan Li, Qi zhu Hong, Zhi jian Hu, Yun li Wu, Xian-E. Peng

**Affiliations:** 1 Department of Epidemiology and Health Statistics, Fujian Provincial Key Laboratory of Environment Factors and Cancer, School of Public Health, Fujian Medical University, Fuzhou, 350108 China; 2 Key Laboratory of Ministry of Education for Gastrointestinal Cancer, Fujian Medical University, Fuzhou, 350108 China

**Keywords:** Nonalcoholic fatty liver disease, Copy number variation, Case control study

## Abstract

**Background:**

The prevalence of Non-alcoholic fatty liver disease (NAFLD) is increasing and emerging as a global health burden. In addition to environmental factors, numerous studies have shown that genetic factors play an important role in the development of NAFLD. Copy number variation (CNV) as a genetic variation plays an important role in the evaluation of disease susceptibility and genetic differences. The aim of the present study was to assess the contribution of CNV to the evaluation of NAFLD in a Chinese population.

**Methods:**

Genome-wide analysis of CNV was performed using high-density comparative genomic hybridisation microarrays (ACGH). To validate the CNV regions, TaqMan real-time quantitative PCR (qPCR) was utilized.

**Results:**

A total of 441 CNVs were identified, including 381 autosomal CNVs and 60 sex chromosome CNVs. By merging overlapping CNVs, a genomic CNV map of NAFLD patients was constructed. A total of 338 autosomal CNVRs were identified, including 275 CNVRs with consistent trends (197 losses and 78 gains) and 63 CNVRs with inconsistent trends. The length of the 338 CNVRs ranged from 5.7 kb to 2.23 Mb, with an average size of 117.44 kb. These CNVRs spanned 39.70 Mb of the genome and accounted for ~ 1.32% of the genome sequence. Through Gene Ontology and genetic pathway analysis, we found evidence that CNVs involving nine genes may be associated with the pathogenesis of NAFLD progression. One of the genes (NLRP4 gene) was selected and verified by quantitative PCR (qPCR) method with large sample size. We found the copy number deletion of NLRP4 was related to the risk of NAFLD.

**Conclusions:**

This study indicate the copy number variation is associated with NAFLD. The copy number deletion of NLRP4 was related to the risk of NAFLD. These results could prove valuable for predicting patients at risk of developing NAFLD.

**Supplementary Information:**

The online version contains supplementary material available at 10.1186/s12876-021-01750-4.

## Introduction

Non-alcoholic fatty liver disease (NAFLD), defined as surplus fat accumulated in the liver, covers a spectrum from simple steatosis to non-alcoholic steatohepatitis (NASH), fatty liver fibrosis (HF), liver cirrhosis (LC) and hepatocellular carcinoma (HCC) [1]. NAFLD is also closely associated with higher prevalence of obesity, type 2 diabetes and cardiovascular disease [2–4]. The prevalence of NAFLD is increasing and emerging as a global health burden, therefore it is crucial to explore pathogenesis and effective prevention management.

In addition to environmental factors, numerous studies have shown that genetic factors play an important role in the development of NAFLD. For example, genome-wide association studies (GWAS) revealed that single-nucleotide polymorphisms of genes are closely related to the risk of NAFLD [5–7]. Copy number variation (CNV) covers submicroscopic variation in the human genome ranging from 1 to 3 Mb in size, and includes insertions, inversions, deletions, duplications and translocations. CNV includes gene coding regions and regulatory elements that can influence gene expression, phenotypic variation and adaptation via disruption of genes, and altering gene dosage [8]. Studies have shown that CNV plays an important role in the evaluation of disease susceptibility and genetic differences in Alzheimer’s disease [9], Parkinson’s disease [10], schizophrenia [11], liver cancer [12] and lung cancer [13]. However, studies on the association between CNV and NAFLD are limited, there exists genetic heterogeneity among study populations, and the sample size is limited.

Herein, a case–control study was designed and conducted in which array-based comparative genomic hybridisation (ACGH) was performed to identify potential CNV associated with NAFLD. Gene annotation analysis software was utilised to ascertain biological processes associated with genes related to CNV. The findings may provide epidemiological evidence for the diagnosis and prevention of NAFLD.

## Materials and methods

### Subjects

The study was approved by local ethics committees of Fujian Medical University (ethics number 2014096). Subjects were recruited from the Health Examination Centre of Nanping First Affiliated Hospital of Fujian Medical University from October 2015 to September 2017. Once cases and controls were linked to NAFLD, a letter of invitation and information about the study was sent to each potential case and control to obtain consent. In order to standardize the experimental process, improve the accuracy of the results, and enhance the comparability of the conclusions, all methods are implemented in accordance with relevant guidelines and regulations.

All subjects underwent abdominal ultrasound, and NAFLD was diagnosed by the presence of at least two of the following three abnormal findings following abdominal ultrasonography [14]: (1) increased echogenicity of the liver near-field region with deep attenuation of the ultrasound signal; (2) hyperechogenity of liver tissue (‘bright liver’), accompanied by hypoechogenicity of the kidney cortex; (3) vascular blurring.

### Clinical and laboratory evaluation

Demographic and anthropometric criteria were assessed for NAFLD patients and normal controls, including sex, age, body mass index (BMI; weight [kg]/height [m^2^]), and waist-to-hip ratio [WHR, waist circumference [cm]/hip circumference [cm]). Various biochemical tests were performed, including fasting blood glucose (FBG), total cholesterol (TC), triglycerides (TG), high-density lipoprotein cholesterol (HDL-C), low-density lipoprotein cholesterol (LDL-C), alanine transaminase (ALT) and aspartate transaminase (AST).

### ACGH and CNV calling

Based on liver fatty accumulation diagnosed by abdominal ultrasound, NAFLD was divided into three grades, and four cases were selected from each grade [15]. The matching principle was formulated according to the gender, age (± 5 years). The controls was selected from healthy people diagnosed with no NAFLD by abdominal ultrasound during the same period. And those who met the case exclusion criteria were not included. Exclusion criteria were as follows: (1) alcohol consumption > 140 g/week for men and > 70 g/week for women; (2) the presence of hepatitis B surface antigens or hepatitis C antibodies; (3) use of hepatotoxic drugs (such as tamoxifen, amiodarone, valproate and methotrexate) that can induce hepatic fat accumulation [16]; (4) hepatic disease, which can induce hepatic fat accumulation; (5) hepatic diseases such as Wilson’s disease, autoimmune hepatitis and hemochromatosis. ACGH was performed on 12 NAFLD patients and 12 healthy controls.

We mainly focus on genetic susceptibility between CNV and NAFLD in the present study, DNA mutations in peripheral leukocytes reflect germline mutation, suggesting the association between mutations and genetic susceptibility, therefore peripheral leukocyte blood was utilised. Genomic DNA was extracted from peripheral blood samples obtained from each subject using a nucleic acid extraction kit (Qiagen, Hilden, Germany). DNA quality was determined by a Denovix DS-11 spectrophotometer (Denovix, Waltham, MA, USA). DNA purity was verified by A260/A280 ratio of 1.80–2.0. ACGH was performed according to the protocol established by the manufacturer (Oxford Gene Technology, Begbroke, UK). It was carried out using SurePrint G3 Human Genome 4 × 180 K microarrays (Agilent Technologies, Santa Clare, CA, USA) for genome-wide identification of putative disease-associated CNVs. The microarrays contained ~ 180,000 probes that enabled the profiling of molecular genomic imbalance with a mean resolution of 13 kb. Probes in the array covered both coding and non-coding regions of the human genome. A total of 1 µg genomic DNA from patients and controls was labelled with Cy3 and Cy5 dyes, respectively and probes were purified and mixed thoroughly using an Agilent SureTag Complete DNA Labeling Kit (Agilent Technologies). This was followed by denaturation and pre-annealing with 50 µg human Cot-1 DNA. Hybridisation of the mixture was performed on an array slide with constant rotation at 65 °C for 40 h. The slide was then washed with Agilent wash buffers 1 and 2, and scanned immediately using an Agilent G2565CA Microarray Scanner (Agilent Technologies). Data were extracted from scanned images using Feature Extraction Software version 10.10 (Agilent Technologies). Raw data were uploaded into Agilent Cytogenomics software (Agilent Technologies). Genomic aberrations were identified by applying log2 intensity ratios of samples to references (Cy3/Cy5, log2 ratios above 0 for duplicates, and below 0 for deletions). CNV was assigned for segments with at least three consecutive probes. Chromosomal aberrations were reported in accordance with the human genome sequence assembly Build 37, hg 19 (http://www.ncbi.nlm.nih.gov).

### Functional enrichment analysis of CNV

After merging overlapping CNVs appearing in two or more samples, a genomic CNV map of NAFLD patients was constructed using R software. Genes associated with CNV were retrieved from the Homo sapiens (GRCh37) assembly provided by University of California Santa Cruz (UCSC). To analyse genes with CNV and investigate the functional impact of CNV on various biological processes, KOBAS gene annotation analysis software which can be accessed at http://kobas.cbi.pku.edu.cn was employed. This program annotates an input set of genes with putative pathways and disease relationships based on mapping to genes with known annotations. It allows for both ID mapping and cross-species sequence similarity mapping. It then performs statistical tests to identify statistically significantly enriched pathways and diseases. KOBAS 2.0 incorporates knowledge across 1327 species from five pathway databases (KEGG PATHWAY, PID, BioCyc, Reactome and Panther) and five human disease databases (OMIM, KEGG DISEASE, FunDO, GAD and NHGRI GWAS Catalog) [17]. The final list of genes associated with NAFLD was determined using the Pubmed database.

### Quantitative PCR validation of CNV calls

To validate the CNV regions, TaqMan real-time quantitative PCR (qPCR) was performed using a Step One Plus instrument (Applied Biosystems) on 557 samples (297 cases and 260 controls) from one selected region (19q13; Assay Hs02992963_cn) which contained the NLRP4 gene. Each reaction (20 µl) contained 10 µl master mix, 1 µl TaqMan Copy Number Assay, 1 ml TaqMan Copy Number Reference Assay, 4 µl nuclease-free water, and 4 µl 5 ng/µl genomic DNA. All reactions were performed in quadruplicate. Thermal cycling conditions consisted of an initial denaturation step at 95 °C for 10 min, followed by 40 cycles at 95 °C for 15 s and 60 °C for 1 min. Furthermore, we analysed CNV in the NLRP4 gene and its association with the risk of NAFLD.

### Statistical analysis

Chi-square tests were employed to assess categorical variables, and Mann–Whitney U tests were used for continuous variables. An unconditional logistical regression model was employed to analyse the association between target gene CNV and NAFLD risk. All statistical analyses were performed by SPSS 23.0 software (SPSS, Inc, Chicago, IL, USA). A two-tailed *p*-value < 0.05 was considered statistical significant.

## Results

### Clinical characteristics of the study population

The clinical characteristics of the study population are outlined in Table 1. Compared with those of the control group, levels of BMI, WHR, ALT, TG, TC, FBG and LDL-C were significantly higher in the NAFLD group (*p* < 0.01–0.05) (Table 1).

**Table 1:** Clinical characteristics of NAFLD patients and normal controls

Characteristics	NAFLD (n = 12)	Controls (n = 12)	*p-value*
Gender (F/M)	9/3	9/3	1
Age (years)	41.33 ± 11.36	41.00 ± 11.33	0.84
WHR	0.92 ± 0.05	0.83 ± 0.04	< 0.001
BMI (kg/m^2^)	26.83 ± 2.78	21.44 ± 1.70	< 0.001
ALT	45 ± 48.20	12.25 ± 4.50	0.001
AST	28.5 ± 18.48	19.5 ± 2.84	0.31
TC	5.81 ± 0.70	4.67 ± 0.28	< 0.001
TG	2.07 ± 1.18	0.82 ± 0.24	< 0.001
FBG	5.57 ± 0.67	4.98 ± 0.33	0.009
LDL-C	3.73 ± 0.63	2.97 ± 0.18	0.001
HDL-C	1.29 ± 0.18	1.33 ± 0.21	0.435

*BMI* body mass index, *WHR* waist-to-hip ratio, *AST* serum aspartate aminotransferase, *ALT* alanine aminotransferase, *GGT* gamma-glutamyltranspeptidase, *FBG* serum fasting glucose, *TC* total cholesterol, *TG* triglycerides, *LDL-C* low-density lipoprotein, *HDL-C* high-density lipoprotein

### Detailed features of CNVRs in the genome

**Fig. 1:**
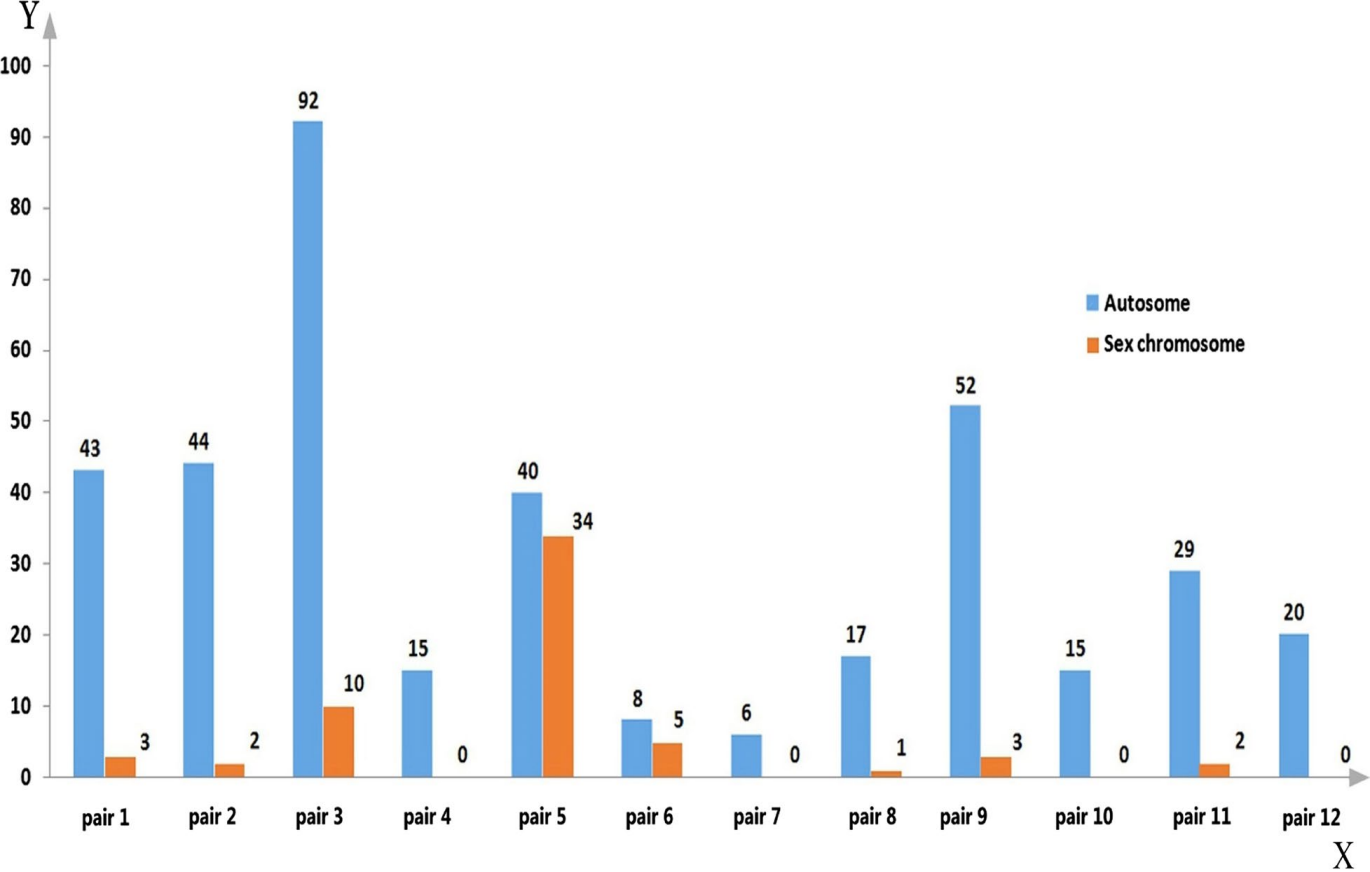
Distribution of genomic CNV in different samples

A rigorous quality control check was performed during sample processing, and all DNA samples were suitable for study. In total, 441 CNVs were detected, including 381 autosomal CNVs and 60 sex chromosome CNVs (Fig. 1). Owing to the evolutionary bias due to small imbalances of sex chromosomes, we opted to exclude sex chromosome CNVs from further analysis. The 381 autosomal CNVs spanned between 5.7 kb and 2.35 Mb in size, with an average size of 181.8 kb (Table 2 and Additional file 1: Table SI). All samples displayed both copy number gains and losses, but copy number gains were more commonly observed than losses (estimated ratio of 1.7:1).

**Table 2:** CNV detected on autosomes of 12 pairs of samples

Sample number	Gains (CNV-containing genes)	Losses (CNV-containing genes)	Range of CNV (kb–Mb)	Total length of CNV (Mb)	Percentage of genome length (%)	Number of genes
Pair 1	40 (26)	3 (3)	15.60–2.35	9.91	0.17	68
Pair 2	6 (4)	38 (32)	7.46–1.93	4.72	0.11	62
Pair 3	5 (4)	87 (52)	7.46–0.46	8.79	0.19	88
Pair 4	9 (8)	6 (5)	5.70–1.17	2.68	0.05	40
Pair 5	1 (1)	39 (31)	8.53–2.23	7.57	3.16	50
Pair 6	4 (4)	4 (2)	27.55–2.08	3.50	0.57	22
Pair 7	2 (2)	4 (4)	20.67–0.46	1.03	0.60	11
Pair 8	12 (1)	5 (3)	10.03–2.35	5.73	0.10	65
Pair 9	6 (6)	46 (34)	7.46–2.03	7.23	0.12	86
Pair 10	8 (7)	7 (6)	9.34–2.13	5.49	0.09	52
Pair 11	13 (11)	16 (14)	17.57–0.85	5.38	0.09	88
Pair 12	11 (10)	9 (6)	13.20–2.27	7.24	0.12	82

**Fig. 2:**
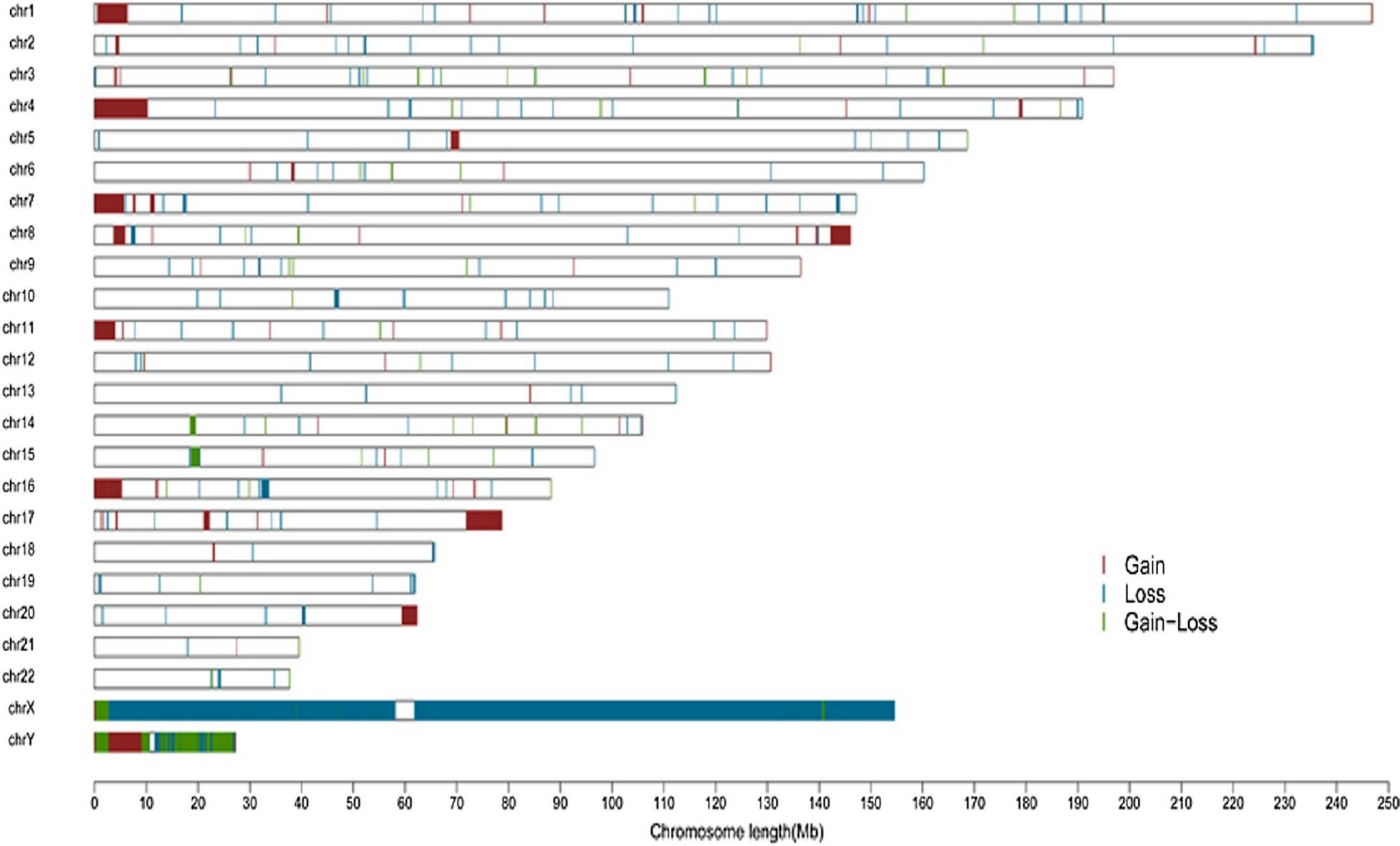
Distribution of CNVRs in the genome

**Fig. 3:**
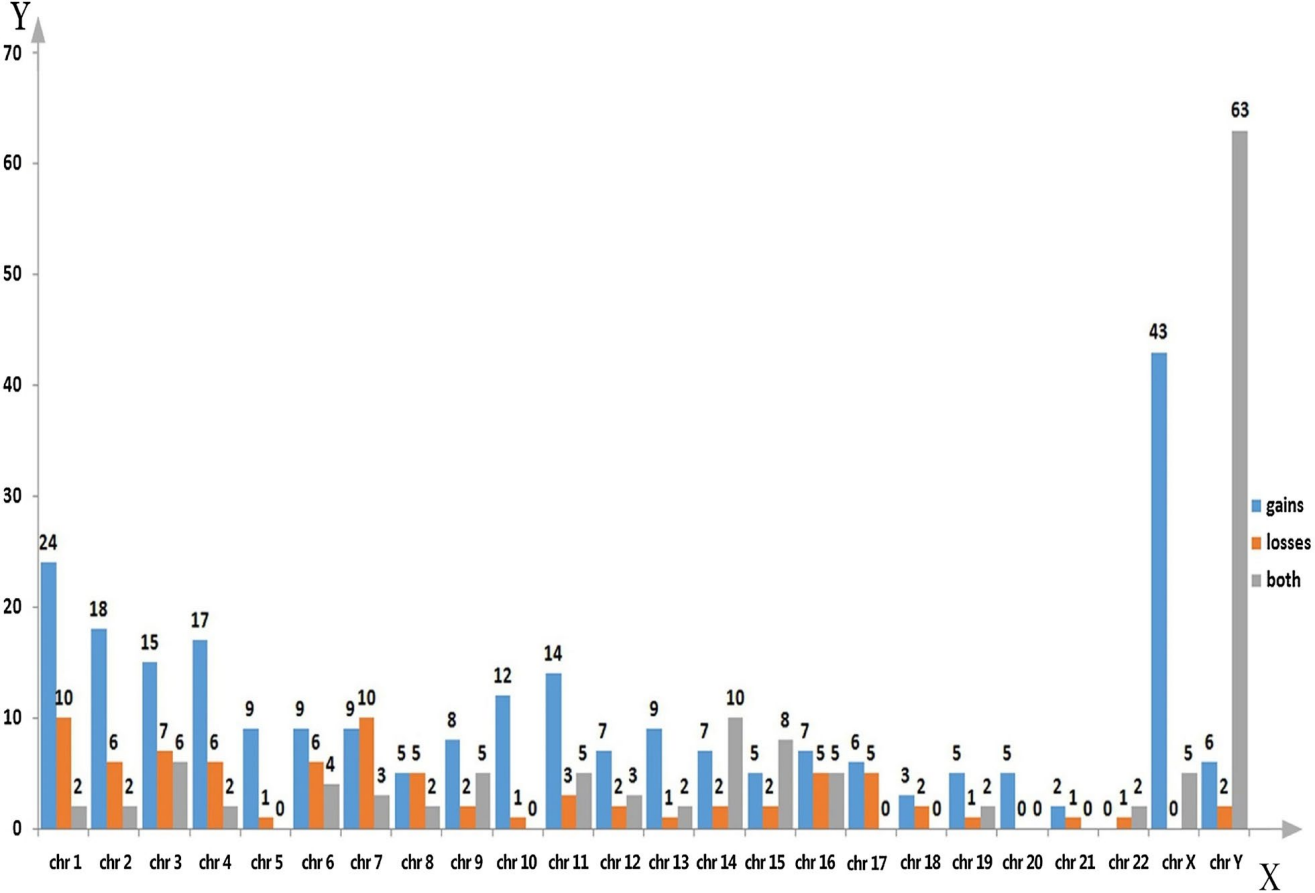
Genomic CNV trends in different samples

Any CNVs overlapping between two or more samples were defined as shared CNVs, and these were integrated, longer fragments were split, and a genomic CNV map of NAFLD patients was constructed using R software. A total of 338 autosomal CNVRs were identified, including 275 CNVRs with consistent trends (197 losses and 78 gains) and 63 CNVRS with inconsistent trends (Figs. 2, 3).

The length of the 338 CNVRs ranged from 5.7 kb to 2.23 Mb, with an average size of 117.44 kb. These CNVRs spanned 39.70 Mb of the genome and accounted for ~ 1.32% of the genome sequence (Table 3). Differences in the number of CNVRs per chromosome were very significant, ranging from 3 for chromosomes 21 and 22 to 36 for chromosome 1. The scales of CNVRs on each chromosome were also different; CNVRs were densely distributed on chromosomes 7, 8, 15 and 16, covering more than 2% of genomic sequences, with CNVRs on chromosome 16 covered 3.33%, compared with only 0.11% for chromosome 20. This demonstrates that the distribution of CNVRs on chromosomes was not uniform.

**Table 3:** Distribution of CNVRs in the genome

Chr	No. of CNVRs (containing genes)	Range of CNV (kb–Mb)	Average CNV (kb)	Total length of CNV (Mb)	Percentage of chromosome length (%)	No. Of genes
1	36 (31)	7.14–0.85	104.26	3.75	1.5	122
2	26 (26)	7.46–0.51	105.01	2.73	1.13	44
3	28 (24)	10.07–0.39	89	2.49	1.26	50
4	25 (25)	9.03–0.58	115.42	2.77	1.46	60
5	10 (10)	17.83–1.52	214.13	1.93	1.06	51
6	19 (19)	8.95–0.44	59.37	1.07	0.63	34
7	22 (19)	8.14–0.69	190.79	4.20	2.64	104
8	12 (12)	15.07–2.23	325.54	3.58	2.47	94
9	15 (11)	7.63–0.20	52.77	0.79	0.57	14
10	13 (11)	13.84–0.73	158.32	2.06	1.54	41
11	22 (22)	5.70–0.28	62.75	1.32	0.98	55
12	12 (12)	8.68–0.2	46.89	0.56	0.42	29
13	12 (8)	17.94–0.35	100.64	1.21	1.06	14
14	19 (15)	9.97–0.47	97.33	1.85	1.73	95
15	15 (15)	15.60–1.49	181.84	2.73	2.68	113
16	17 (14)	25.82–0.8	176.96	3.01	3.33	139
17	11 (11)	18.61–0.41	96.05	1.06	1.27	64
18	5 (5)	49.08–0.31	125.72	0.63	0.78	13
19	8 (8)	16.49–0.42	107.02	0.86	1.47	56
20	5 (4)	12–24.69	18.14	0.07	0.11	8
21	3 (2)	0.98–0.11	41.48	0.12	0.26	4
22	3 (3)	26.37–0.29	119.47	0.36	0.71	21
X	48 (40)	17.85–31.52	3143.22	150.87	96.69	2357
y	71 (62)	12.82–3.96	357.95	25.41	44.40	506

### Gene content of genomic CNVs and identification of candidate genes associated with NAFLD by functional enrichment.

A total of 1225 genes were annotated in 307 autosomal CNV regions by human genome sequence assembly using Build 37, hg19 (http://www.ncbi.nlm.nih.gov). Functional analysis clustering of these genes was performed using KOBAS gene annotation analysis software, and genes associated with NAFLD were identified using the Pubmed database. Finally, we identified nine CNVRs related to NAFLD (chr3, 57199594-57590187; chr7, 54813380-55274871; chr17, 38688567-38738474; chr19, 56370486-56416408; chr22, 24347959-24390254; chr14, 74001651-74022324; chr12, 57897795-57918452; chr1, 65935075-65959904; chr20, 33470663-33495348) corresponding to genes APPL1, EGFR, CCR7, NLRP4, GSTT1, ACOT1, CHOP, LEPR and ACCS2 (Table 4). By regulating lipid metabolism enzyme activity, adiponectin, insulin signalling, immune cell activity, inflammatory mediator levels and cell phase II response, these genes are closely associated with NAFLD.

**Table 4:** CNV region associated with NAFLD

Region	Gene	Sample frequency (gains, losses)	Function	GO item	Relevant study ( DOI)
Chr3:57199594-57590187	APPL1	1 (0, 1)	Adiponectin, insulin signalling	GO:0008286	10.1371/journal.pone.0071391
Chr7:54813380-55274871	EGFR	1 (0, 1)	Tyrosine phosphorylation regulates cell proliferation	GO:0000165	10.3969/j.issn.1674-4136.2017.05.016; 10.1016/j.bbadis.2017.10.016
Chr17:38688567-38738474	CCR7	2 (0, 2)	Participates inflammatory response	GO:0007249	10.1038/ijo.2017.200; 10.3870/j.issn.1672-0741.2017.06.011
Chr19:56370486-56416408	NLRP4	3 (0, 3)	Inhibition of type I interferon and NF-KB inflammatory signalling	GO:0032479	10.1186/s12876-014-0208-8; 10.1074/jbc.M200446200
Chr22:24347959-24390254	GSTT1	7 (6, 1)	Participates in the phase II reaction and promote toxic metabolism	GO:0004602	10.1016/j.clinre.2011.01.015
Chr14:74001651-74022324	ACOT1	2 (1, 1)	Regulation of lipid metabolism enzyme activity	GO:0071616	10.2337/db16-1519; 10.1194/jlr.M081455
Chr12:57897795-57918452	CHOP	1 (1, 0)	Participates in endoplasmic reticulum stress and promote apoptosis	GO:0036488	10.1038/labinvest.2016.61; 10.1016/j.ejps.2011.02.005
Chr1:65935075-65959904	LEPR	1 (0, 1)	Regulates leptin signalling	GO:0038021	10.2174/1566524018666180705110412; 10.3870/lcxh.j.issn.1005-541X.2011.03.07
Chr20:33470663-33495348	ACSS2	1 (0, 1)	Regulation of lipid metabolism enzyme activity	GO:0006085	10.1073/pnas.1806635115; 10.1002/jcp.25954

### Validation of CNV by qPCR

The results of ACGH showed that 3 out of 12 pairs harboured copy number losses in chr19 (56370486-56416408) which contained the NLRP4 gene. Functional enrichment analysis indicated that this gene may participate in inflammatory responses by regulating the levels of inflammatory mediators.

Since inflammatory factors play an important role in the development of NAFLD, quantitative PCR (qPCR) with large sample size was performed to validate this CNV. A total of 557 subjects were included in the study, comprising 297 cases and 260 controls. In case group, the prevalence of obesity and hypertension was significantly higher than that in control group. The level of ALT, AST, FBG and TG were higher in the control group, and the LDL-C was higher in cases (Tables 5, 6).

**Table 5:** Baseline characteristics of the study population

	Cases (%)	Controls (%)	χ^2^/Z	*p value*
*Gender*				
Male	209 (70.4)	185 (71.2)	0.04	0.84
Female	88 (29.6)	75 (28.8)		
Age (years)	45.66 ± 11.764	45.48 ± 11.804	− 0.16	0.87
*Education*				
Primary education	63 (21.2)	55 (21.2)	1.97	0.37
Secondary education	88 (29.6)	64 (24.6)		
Bachelor degree	146 (49.2)	141 (54.2)		
*Marital status*				
Single	31 (10.4)	36 (13.8)	1.52	0.22
Married or divorced	266 (89.6)	224 (86.2)		
*Income (¥)*				
< 1000	16 (5.4)	15 (5.8)	0.36	0.83
1000–2000	83 (27.9)	78 (30)		
≥ 2000	198 (66.7)	167 (64.2)		
*BMI (kg/m*^*2*^*)*				
< 18.5	2 (0.7)	9 (3.5)	117.95	**< 0.001**
18.5–23.9	99 (33.3)	193 (74.2)		
24.0–27.9	149 (50.2)	57 (21.9)		
≥ 28	47 (15.8)	1 (0.4)		
*Blood pressure (mm/Hg)*				
< 140/90	211 (71)	211 (81.2)	7.72	**0.01**
≥ 140/90	86 (29)	49 (18.8)		
*Diabetes*				
Yes	12 (4)	7 (97.3)	0.77	0.38
No	285 (96)	253 (2.7)		
*Tea consumption*				
No	112 (37.7)	109 (41.9)	1.03	0.311
Yes	185 (62.3)	151 (58.1)		
*Smoking habit*				
No	220 (74.1)	197 (75.8)	0.21	0.65
Yes	77 (25.9)	63 (24.2)		

Bold indicates that the analysis result is statistically significant

**Table 6:** Clinical characteristics of NAFLD patients and normal controls

Characteristics	Cases (%)	Controls (%)	*Z*	*p value*
ALT (IU/l)	27 (19–38)	19 (14–27)	− 7.91	**< 0.001**
AST (IU/l)	23 (20–28)	20 (18–24)	− 5.59	**< 0.001**
GLU (mmol/l)	5.34 (4.97–5.87)	5.19 (4.91–5.52)	− 3.34	**0.001**
TC (mmol/l)	5.09 (4.49–5.65)	5.03 (4.56–5.45)	− 0.94	0.35
TG (mmol/l)	1.77 (1.26–2.51)	1.18 (0.87–1.55)	− 9.50	**< 0.001**
HDL (mmol/l)	3.13 (2.53–3.75)	3.19 (2.687–3.56)	− 0.49	0.62
LDL (mmol/l)	1.21 (1.06–1.37)	1.35 (1.20–1.47)	− 6.70	**< 0.001**

Bold indicates that the analysis result is statistically significant

*AST* serum aspartate aminotransferase, *ALT* alanine aminotransferase, *GGT* gamma-glutamyltranspeptidase, *FBG* serum fasting glucose, *TC* total cholesterol, *TG* triglycerides, *LDL-C* low-density lipoprotein, *HDL-C* high-density lipoprotein

**Fig. 4:**
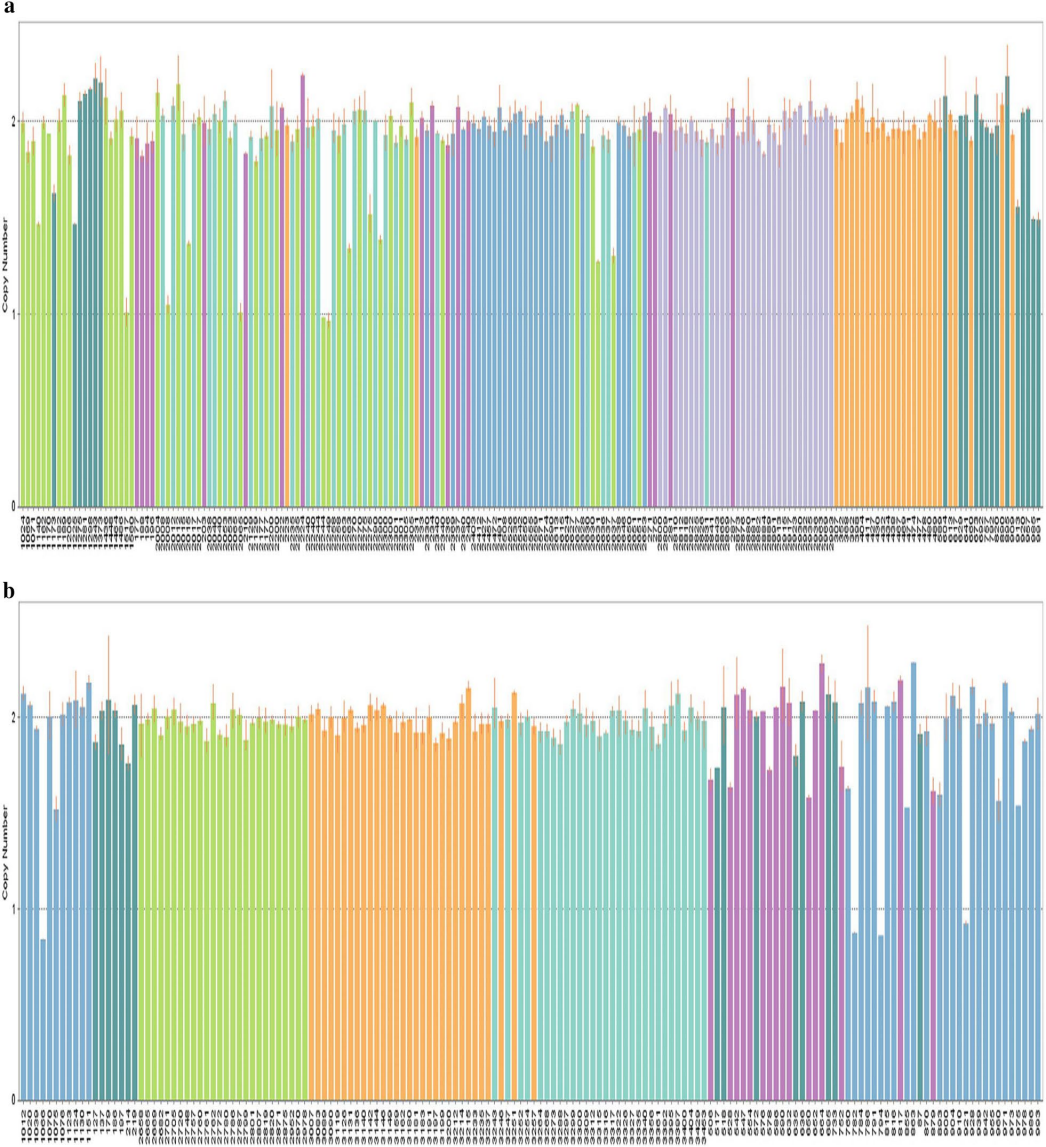

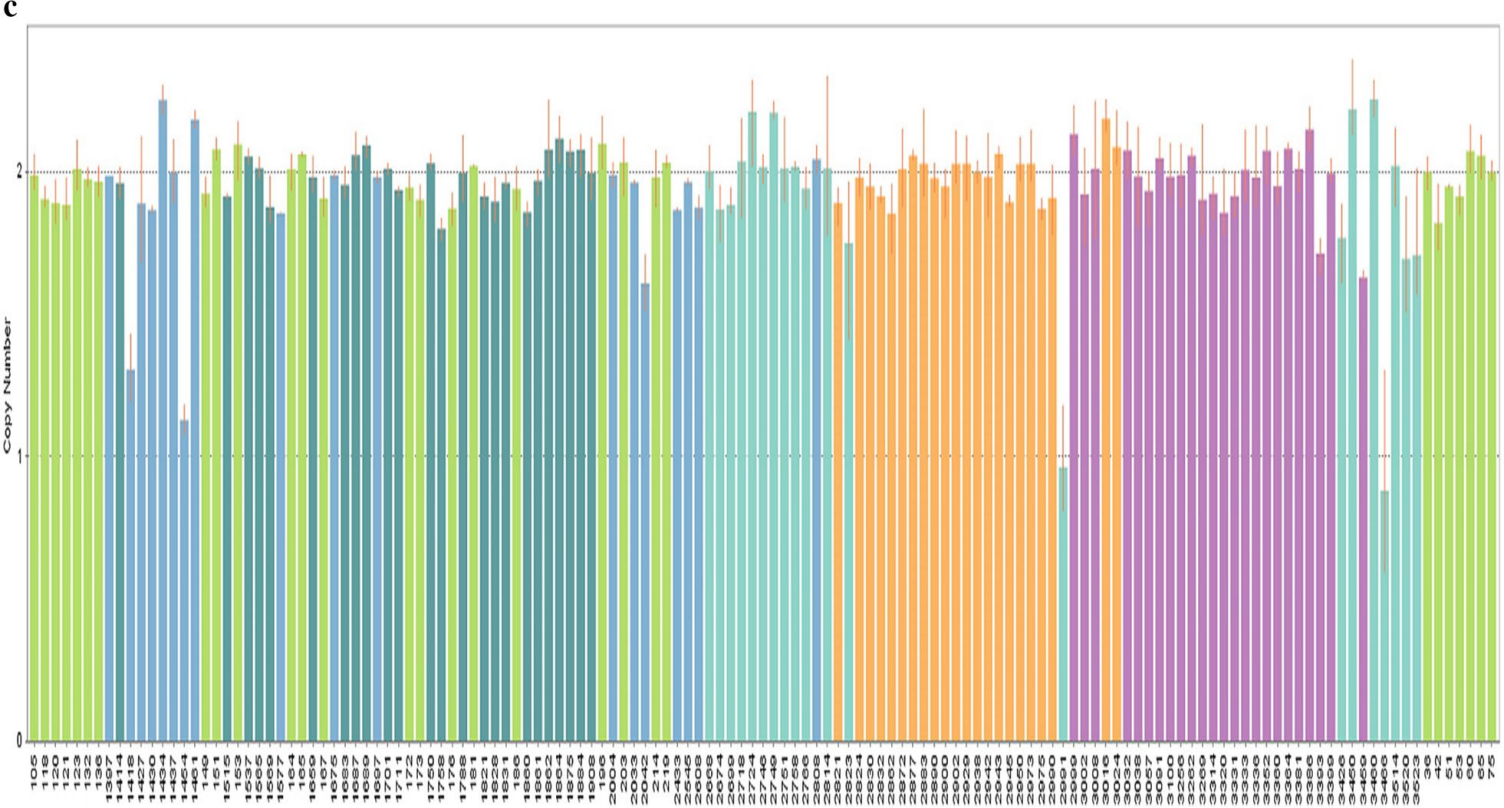
Copy number distribution of the NLRP4 gene (Because the copy number analysis software (copy caller) has a limit on the number of samples in each analysis, all the experimental samples were randomly grouped and analyzed in three times, and finally three figures were obtained: Figure a, b, c)

Copy numbers were calculated using CopyCallerv2.0 software and categorised as losses (< 2), normal (= 2), and gains (> 2). In the present work, the NLRP4 gene copy number distribution ranged from 1 to 2, with 15 (5.1%) losses and 282 (94.9%) normal copy number in cases. There were four (1.5%) losses and 256 (98.5%) normal copy number in controls. The distribution of NLRP4 gene copy number variation was statistically significant between the two groups (χ^2^ = 5.19, *p* = 0.02; Table 7, Fig. 4a, b, c). Furthermore, to investigate the relationship between NLRP4 gene copy number variation and the risk of NAFLD, unconditional logistical regression analysis was performed. The results showed that NLRP4 gene copy number deletion was associated with the risk of NAFLD (OR = 3.40, 95% *CI* = 1.12–10.39). After adjustment for confounding factors (gender, age, BMI, blood pressure and diabetes history), the association was still statistically significant (OR = 4.49, 95% *CI* = 1.3–15.52; Table 7).

**Table 7:** Relationship between NLRP4 gene copy number variation and the risk of NAFLD

Group	One copy	Two copies	*χ*^2^ (*p*)	*OR* (95% *CI*)	
				Model 1	Model 2
Cases	15 (5.1)	282 (94.9)	5.19 (0.02)	3.40 (1.12–10.39)	4.49 (1.3–15.52)
Controls	4 (1.5)	256 (98.5)			

Model 1: unadjusted model

Model 2: adjusted for gender, age, blood pressure, BMI, Diabetes history

## Discussion

In this study, we identified 338 autosomal CNV regions, with 1225 genes annotated in 307 of these CNV regions. Through gene enrichment analysis and a review of the existing literature and Pubmed database, we identified nine CNV regions associated with the development of NAFLD, including chr2 (24347959-24390254, GSTT1), Chr14 (74001651-74022324, ACOT1), chr20 (33470663-33495348, ACSS2), Chr7 (54813380-55274871, EGFR), chr1 (65935075-65959904, LEPR), Chr3 (57199594-57590187, APPL1), chr12 (57897795-57918452, CHOP), chr19 (56370486-56416408, NLRP4) and chr17 (38688567-38738474, CCR7).

Seven samples exhibited CNV in the region of Chr22 (24347959-24390254) which contained the glutathione-s-transferase 1 gene (GSTT1). Six samples displayed copy number gains, and one sample displayed copy number losses. GSTs are a superfamily of proteins that participate in phase II detoxification, which promotes the elimination of toxic metabolites and reduces oxidative damage by catalysing the conjugated binding of glutathione to electrophilic substances (such as peroxides, superoxide ions, lipid peroxides, etc.). Studies have shown the GSTT1 gene polymorphism is closely related to the risk of metabolic diseases such as coronary heart disease [18] and diabetes [19]. However, one study targeted at Southeast Iran showed that genetic polymorphism of GSTM1 and GSTP1, but not GSTT1, was associated with NAFLD [20].

The CNV regions Chr14:74001651-74022324 and chr20:33470663-33495348 contained the genes acyl-CoA thioesterase 1 (ACOT1) and acyl-CoA synthetase short-chain family member 2 (ACSS2), both of which are related to lipid metabolism. ACOT1 is a key cytosolic enzyme that participates in fatty acid (FA) metabolism by catalysing the conversion of acyl-CoAs to FAs and coenzyme A, and it is also believed to be a target gene of PPARα [21–23]. Previous studies revealed that overexpression of ACOT1 reduces the availability of excess long-chain acyl-CoAs for β-oxidation, and represses PPARα signalling pathways to reverse altered substrate metabolism and attenuate increased oxidative stress, mitochondrial dysfunction, and cardiac inefficiency in diabetic hearts [24]. In mice fed a high fat diet, Acot1 knockdown elicits a robust induction of inflammatory and oxidative stress markers, and increased ROS levels. Thus, when a high fat diet induces steatosis, ACOT1 protects against inflammation and oxidative stress that lead to fibrosis [25].

ACSS2 plays a key role in lipogenesis by converting acetate to acetyl-CoA for lipogenesis [26]. A recent study showed that knockdown of ACSS2 increased the invasion and migration abilities of HCC cells, which plays an important role in the prognosis of patients with HCC [27]. Another study also found that mRNA levels of genes associated with de novo fatty acid synthesis, triacylglycerol synthesis, lipid droplet formation and fatty acid oxidation were downregulated after ACSS2 knockdown [28]. In diet-induced obese mice, lack of ACSS2 results in a significant reduction in body weight and hepatic steatosis [29].

The CNV region chr1:65935075-65959904 contains the gene encoding leptin receptor (LEPR) that binds to leptin in target tissues. Intravenous administration of leptin reduces appetite, while its deficiency increases food intake. Furthermore, leptin can modulate insulin secretion and action through LEPR, and polymorphism of LEPR has been linked to NAFLD [30].

The CNV region Chr3:57199594-57590187 contains genes encoding Adaptor protein and PH domain- and leucine zipper-containing 1 (APPL1). APPL1 is the first identified adaptor protein that interacts directly with adiponectin receptors. Adiponectin is a peptide secreted by adipocytes that plays an important role in regulating glucose and lipid metabolism, and controlling inflammation in insulin-sensitive tissues. Studies have linked low adiponectin levels to NAFLD. Adiponectin signalling through APPL1 is necessary to exert its anti-inflammatory and cytoprotective effects on endothelial cells. APPL1 also functions in insulin signalling pathways, it is an important mediator of adiponectin-dependent insulin sensitisation in skeletal muscle, and it acts as a mediator of other signalling pathways by interacting directly with membrane receptors or signalling proteins. Thus, APPL1 plays critical roles in cell proliferation, apoptosis, cell survival, endosomal trafficking and chromatin remodelling [31]. Polymorphism of APPL1 has been associated with NAFLD [32].

The CNV region chr19:56370486-56416408 contains the gene encoding NLRP4, a negative regulator of pro-inflammatory cytokines that is associated with inactivation of IKKa/NF-kB [33]. A previous study demonstrated that NLRP4 attenuates the inflammation response by reducing the expression of transforming growth factor-β1 (TGF-β1), tumour necrosis factor-α (TNF-α), interleukin-1β (IL-1β), IL-18 and IL-6 in fructose-treated cardiac cells [34]. In visceral adipose tissue from patients with pericellular fibrosis, NLRP4 mRNA levels were significantly lower than in those without pericellular fibrosis, and the NLRP 4 gene is significantly downregulated in adipose tissue from NASH patients [35].

The CNV region chr17:38688567-38738474 contains the gene encoding C–C chemokine receptor 7 (CCR7), which is primarily expressed in immune cells. Studies found that CCR7 deficiency leads to the development of multi-organ autoimmunity [36], chronic renal disease [37] and autoimmune diabetes [38]. A recent study showed that infiltration of CCR7-expressing cells in adipose tissue is associated with insulin resistance in obesity [39]. The absence of CCR7 decreases IL-10-producing iNKT cells in fatty liver, and exacerbates NAFLD [40].

The CNV region Chr7:54813380-55274871 contains the gene encoding epidermal growth factor receptor (EGFR), a receptor tyrosine kinase expressed in activated hepatic stellate cells (HSCs) that is associated with the development of liver fibrosis. One study indicated that depressing the phosphorylation of EGFR can reduce the number of activated HSCs [41]. Another study suggested that EGFR is phosphorylated in liver tissues in a high-fat diet-induced murine model of NAFLD. Inhibition of EGFR prevents diet-induced lipid accumulation, oxidative stress, HSC activation and matrix deposition [42].

The CNV region chr12:57897795-57918452 contains the gene encoding CCAAT/enhancer-binding protein (C/EBP) homologous protein (CHOP), a major transcriptional regulator of endoplasmic reticulum (ER) stress-mediated apoptosis that is implicated in lipotoxicity-induced ER stress and hepatocyte apoptosis in NAFLD [43]. A previous study found that CHOP knockout (CHOP-/-) mice fed a high-fat diet developed more severe histological NASH features than wild-type controls;The severity of NASH in high-fat diet-fed CHOP−/− mice correlated with a significant decrease in peroxisomal β-oxidation, increased *denovo* lipogenesis, and ER stress-mediated hepatocyte apoptosis [44]. These findings indicate that CHOP protects hepatocytes from ER stress, and plays a significant role in the mechanism of liraglutide-mediated protection against NASH pathogenesis.

The use of ACGH technology allows CNV discovery at high resolution, and hence confidence in CNV detection. Therefore, this approach may be used to identify noninvasive biomarkers with potential for the pathological evaluation of NAFLD. However, as more comprehensive studies are still ongoing, genes known to be related to NAFLD will be investigated, and our preliminary results remain to be substantiated by studies on larger patient groups. In addition, functional studies on genes residing within these loci are needed to fully characterise the functions of genes and their relationships with NAFLD.

## Conclusions

In case group, the copy number deletion of NLRP4 gene in the Chr19:56370486-56416408 region was higher than that in control group. The copy number deletion of this gene was related to the risk of NAFLD.

## Supplementary Information

**Additional file 1.** CNV detected on sex chromosomes of 12 pairs of samples.

## Data Availability

All data generated or analyzed during this study are included in this published article or are available from the corresponding author on reasonable request.

## Abbreviations

ACGH: Array⁃based comparative genomic hybridization
AST: Asparagine aminnotransferase
ALT: Alanine aminotransferase
BMI: Body mass index
CNV: Copy number variation
FBG: Fasting blood glucose
GGT: γ-Glutamyl transferase
HDL-C: HDL cholesterol
IR: Insulin resistance
LDL-C: LDL cholesterol
NAFLD: Non-alcoholic fatty liver disease
NLRP4: NLR family pyrin domain containing 4
OR: Odds ratio
TC: Total cholesterol
TG: Triglycerides
