# Supplementary material for: Identifying potential biomarkers of nonalcoholic fatty liver disease via genome-wide analysis of copy number variation
Source: BMC Gastroenterol. 2021 Apr 14;21:171. doi: 10.1186/s12876-021-01750-4 (PMC8045212; doi:10.1186/s12876-021-01750-4)
Supplement: Supplementary file 1 — Additional file 1. CNV detected on sex chromosomes of 12 pairs of samples. [file 12876_2021_1750_MOESM1_ESM.doc]

**Identifying potential biomarkers of nonalcoholic fatty liver disease via genome-wide analysis of copy number variation**

Yang fan Li^1#^, Jing Zheng^1#^, He wei Peng^1^, Xiao lin Cai^1^, Xin ting Pan^1^, Hui quan Li^1^, Qi zhu Hong^1^, Zhi jian Hu^1^, Yun li Wu^2^**^*^**, Xian-E Peng^1, 2^**^*^**

^1^Department of Epidemiology and Health Statistics, Fujian Provincial Key Laboratory of Environment Factors and Cancer, School of Public Health, Fujian Medical University, Fuzhou 350108, China.

^2^Key Laboratory of Ministry of Education for Gastrointestinal Cancer, Fujian Medical University, Fuzhou 350108, China.

^#^contributed equally

* Corresponding author.

Xian-E Peng, Department of Epidemiology and Health Statistics, Fujian Provincial Key Laboratory of Environment Factors and Cancer, School of Public Health, Fujian Medical University, Fuzhou 350108, China

Phone: +86-591 22862648 mail: [fmuxe@163.com](mailto:fmuxe@163.com)

Yun li Wu, Key Laboratory of Ministry of Education for Gastrointestinal Cancer, Fujian Medical University, Fuzhou 350108, China

Phone: +86-591 22862648 mail: [wuyinli422@163.com](mailto:fmuxe@163.com)

**Running title:** Li yf, et al: Identifying biomarkers of nonalcoholic fatty liver disease

| **Table SI CNV detected on sex chromosomes of 12 pairs of samples** | | | | | | |
| --- | --- | --- | --- | --- | --- | --- |
| Sample number | Copy number gains (containing genes) | Copy number losses (containing genes) | Range of CNV (kb−Mb) | Total length of CNV (Mb) | Percentage of genome length (%) | Number of genes |
| Pair 1 | 3 (1) | 0 **(**0) | 57.43−0.09 | 0.22 | 0.17 | 1 |
| Pair 2 | 0 (0) | 2 **(**2) | 25.10−1.64 | 1.67 | 0.11 | 6 |
| Pair 3 | 0 (0) | 10 **(**4) | 17.85−1.49 | 2.34 | 0.19 | 16 |
| Pair 4 | 1 (0) | 1 **(**1) | 33.32−0.24 | 0.27 | 0.05 | 7 |
| Pair 5 | 30 (16 | 4 **(**4) | 20.78−93 | 181.97 | 3.16 | 1105 |
| Pair 6 | 1 (1) | 4 **(**4) | 494.66−11.29 | 30.68 | 0.57 | 145 |
| Pair 7 | 0 (0) | 4 **(**4) | 2.50−15.29 | 34.80 | 0.60 | 166 |
| Pair 8 | 0 (0) | 1 **(**1) | 88.84 | 88.84 | 0.10 | 1 |
| Pair 9 | 0 (0) | 3 **(**1) | 46.76−0.10 | 0.21 | 0.12 | 2 |
| Pair 10 | 0 (0) | 0 **(**0) | 0 | 0 | 0.09 | 0 |
| Pair 11 | 0 (0) | 2 **(**1) | 20.84−53.24 | 0.07 | 1 | 1 |
| Pair 12 | 0 (0) | 0 **(**0) | 0 | 0 | 0.12 | 0 |
